# Essential oil from *Piper aduncum* L. leaves exhibits analgesic and anti-inflammatory activity in mice

**DOI:** 10.1007/s10787-026-02415-y

**Published:** 2026-10-07

**Authors:** Paulo Henrique Eloi Fernandes, Paulo Henrique Andrade do Nascimento Silva, Suyane de Deus e Melo, Bruna de Sousa Gomes, Ryan Cristian da Silva, Laís Ruanita Leopoldina Galvão, Bruno Vinicius Souza da Silva, Maria Clara Morais Cavalcanti, Rayssa Nilma da Silva Alves, Yasmin Antonelle Correia Lira, Júlio César Ribeiro de Oliveira Farias de Aguiar, Daniela Maria do Amaral Ferraz Navarro, Maria Tereza dos Santos Correia, Wêndeo Kennedy Costa, Márcia Vanusa da Silva

**Affiliations:** 1 Department of Biochemistry, Biosciences Center, Federal University of Pernambuco (UFPE), Recife, Pernambuco 50670-420 Brazil; 2 Chemical Ecology Laboratory, Department of Fundamental Chemistry, Federal University of Pernambuco, Recife, PE 50740-560 Brazil

**Keywords:** Antinociception, Antipyretic activity, ATP-Sensitive Potassium Channels, Dilapiol, Inflammation, Muscarinic receptors, Pipeaceae

## Abstract

*Piper aduncum* L. is a medicinal plant whose essential oil is rich in bioactive constituents, particularly dilapiol, and has attracted interest because of its diverse pharmacological properties. However, its anti-inflammatory, antipyretic, and antinociceptive activities and the mechanisms potentially underlying these effects remain insufficiently characterized. Therefore, this study investigated the pharmacological effects of *P. aduncum* leaf essential oil (EO*Pa*) and explored potential mechanisms involved in its antinociceptive activity. EO*Pa* was obtained by hydrodistillation and chemically characterized by GC–MS and GC–FID. Its pharmacological effects were evaluated in male Swiss mice using brewer’s yeast-induced pyrexia, carrageenan-induced paw edema, acetic acid-induced writhing, and the formalin test. Potential mechanisms of antinociceptive activity were investigated using naloxone, atropine, L-NAME, and glibenclamide as pharmacological antagonists/inhibitors. Chemical characterization identified dilapiol as the major constituent of EOPa (69.65 ± 2.12%). EOPa showed dose-dependent anti-inflammatory, antipyretic, and antinociceptive effects, with the most pronounced activity observed at 200 mg/kg. The essential oil reduced inflammatory edema, fever, and nociceptive responses in the experimental models. EOPa at 200 mg/kg reduced yeast-induced fever by up to 76.1% (*p* < 0.01), carrageenan-induced paw edema by up to 26.48% (*p* < 0.0001), and acetic acid-induced writhing by 65.90% (*p* < 0.001). In the formalin test, EO*Pa* reduced nociceptive behavior by 39.89% during the neurogenic phase and by 70.67% during the inflammatory phase (*p* < 0.0001). Pretreatment with atropine and glibenclamide markedly attenuated the antinociceptive effect of EO*Pa*, whereas naloxone and L-NAME did not significantly modify the response. These findings provide preclinical evidence that P. aduncum leaf essential oil possesses anti-inflammatory, antipyretic, and antinociceptive activities and suggest that the antinociceptive effect of EOPa involves activation of muscarinic receptors, consistent with a muscarinic agonist-like effect, and stimulation of ATP-sensitive potassium (K^+^_ATP_) channels, consistent with a K^+^_ATP_-channel-stimulating effect. Further pharmacological, toxicological, and mechanistic studies are warranted to clarify the molecular basis, active constituents, and safety profile of these effects.

**Graphical abstract:**

**Figure:**
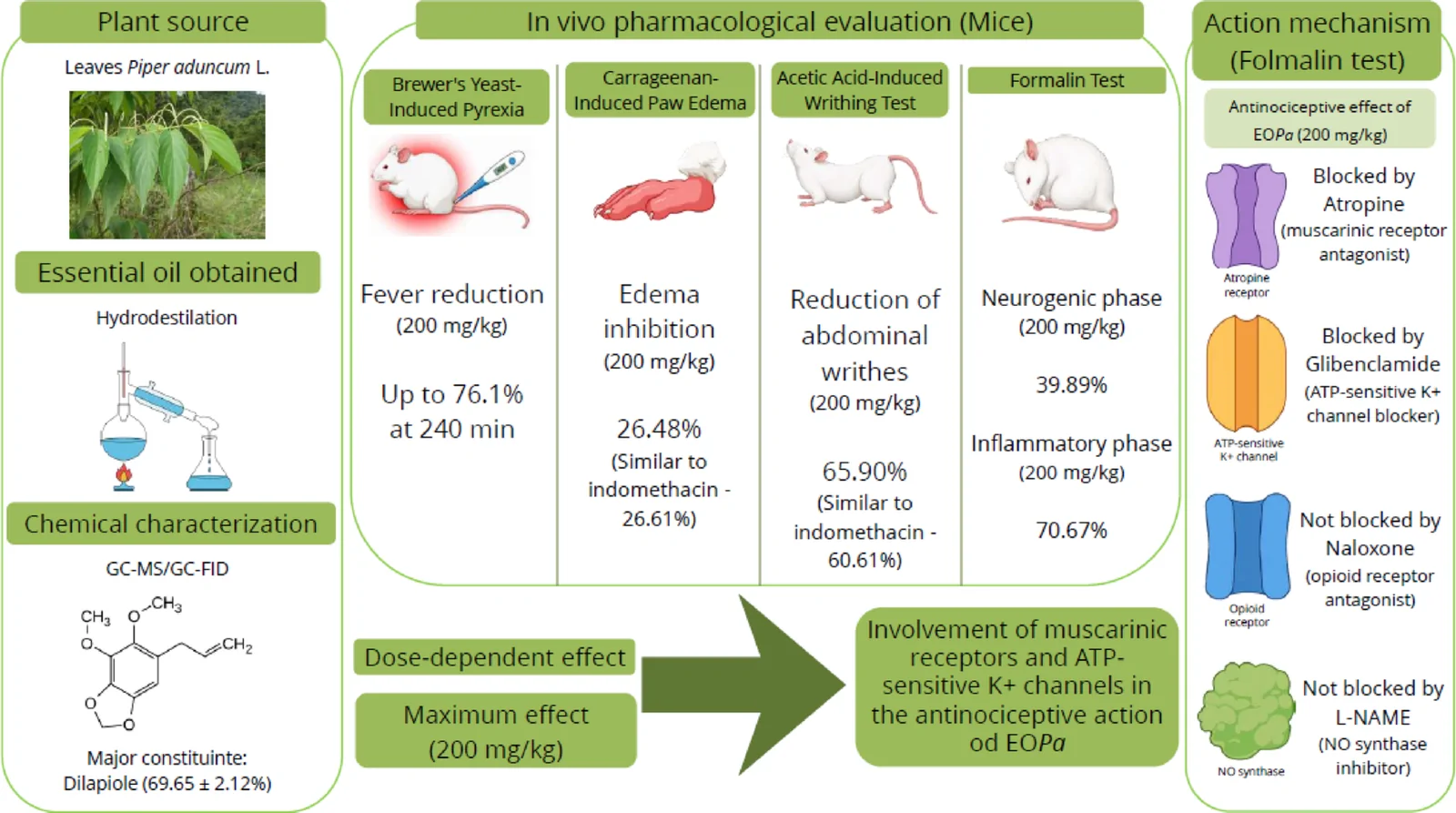

## Highlights

- *Piper aduncum* essential oil showed dose-dependent anti-inflammatory, antipyretic, and antinociceptive effects in vivo.
- *Piper aduncum* essential oil showed dose-dependent anti-inflammatory, antipyretic, and antinociceptive effects in vivo.
- Reduced writhing (up to 65.90%) and formalin pain response (up to 70.67%).
- Decreased carrageenan-induced paw edema by up to 26.48%, comparable to indomethacin.
- Lowered yeast-induced fever by up to 76.1% at 200 mg/kg.
- Effects involve muscarinic receptors and ATP-sensitive K^+^ channels.

## Introduction

Inflammation is an essential physiological response that enables the body to combat infections, repair tissue damage, and maintain homeostasis. This dynamic process is tightly regulated to ensure an effective response to tissue injury or pathogenic challenges while minimizing damage to host tissues (Mehta 2025). However, disruption of this regulatory balance may lead to excessive or persistent inflammation, contributing to the pathogenesis of several disorders, including cardiovascular diseases, autoimmune conditions, metabolic disorders, and cancer (Dhurandhar et al. 2025). Because inflammation is closely associated with pain and fever, the identification of pharmacological agents capable of modulating these responses remains an important objective in biomedical research (Sun et al. 2025; Calatroni et al. 2025).

Medicinal plants have long been used in the prevention and treatment of human diseases and remain important sources of bioactive substances with diverse pharmacological properties (Kujawska and Albán-Castillo 2025). Among plant-derived preparations, essential oils have attracted considerable scientific interest because of their chemically complex composition and broad biological activities. These volatile mixtures contain predominantly terpenoids, particularly monoterpenes and sesquiterpenes, as well as other classes of constituents, and are commonly obtained by steam distillation or hydrodistillation (Shiekh et al., 2024). Their pharmacological effects may result from the activity of individual constituents as well as from interactions among multiple compounds, making the characterization of both chemical composition and biological effects important for evaluating their therapeutic potential.

The family Piperaceae comprises a diverse group of plants widely distributed in tropical and subtropical regions. Within this family, the genus Piper includes numerous species of ethnopharmacological and pharmacological relevance, whose chemical diversity has been associated with a broad range of biological activities (Simmonds et al. 2021; Takooree et al. 2019). Among these species, *Piper aduncum* L. is an abundant source of essential oils with promising pharmacological potential and is widely distributed throughout tropical and subtropical ecosystems.

Previous studies have reported several biological activities associated with *P. aduncum* essential oil, including analgesic and anti-inflammatory effects, as well as antidepressant, anxiolytic, and gastroprotective activities (Alvarado-García et al. 2024; Eloi Fernandes et al. 2026). The species has also demonstrated antiparasitic properties, including antiprotozoal and anthelmintic effects (Monzote et al. 2017; Oliveira et al. 2014). In addition, dilapiol, a major constituent commonly found in *P. aduncum* essential oil, has been associated with anti-inflammatory activity, further supporting the pharmacological relevance of this species (Eloi Fernandes et al. 2026).

Despite these findings, important aspects of the pharmacological profile of *P. aduncum* leaf essential oil remain insufficiently characterized. Previous studies have reported biological and pharmacological activities of the essential oil, including anti-inflammatory and antinociceptive effects, but the available evidence remains limited and heterogeneous with respect to experimental models, plant origin, and chemical composition (Guerrini et al. 2009). In particular, the pharmacological profile of the complete essential oil has not been comprehensively characterized across complementary models addressing inflammation, fever, and different components of nociceptive processing. Moreover, although biological activities have been investigated for individual constituents of *P. aduncum* essential oil, particularly dillapiole, the mechanisms underlying the effects of the complete phytocomplex remain insufficiently understood. Therefore, further investigation using complementary pharmacological models and mechanistic approaches is warranted to better characterize the breadth of the pharmacological activity of *P. aduncum* leaf essential oil and to identify potential pathways involved in its antinociceptive effects.

Therefore, the present study was designed to provide an integrated in vivo pharmacological characterization of *P. aduncum* leaf essential oil, evaluating its anti-inflammatory, antipyretic, and antinociceptive effects in complementary experimental models. The study assessed carrageenan-induced paw edema, brewer’s yeast-induced pyrexia, acetic acid-induced writhing, and both neurogenic and inflammatory phases of the formalin test. In addition, pharmacological antagonism was used to investigate the potential involvement of opioid and muscarinic receptors, nitric oxide signaling, and ATP-sensitive potassium (K_ATP_) channels in the antinociceptive effects of the essential oil. This approach extends previous evidence regarding the biological activity of *P. aduncum* essential oil by providing a broader pharmacological profile and new mechanistic evidence suggesting the involvement of muscarinic receptors and K_ATP_ channels in its antinociceptive effects.

## Methodology

### Plant material collection

Leaves of *Piper aduncum* L. were collected from a forested area located in Curado, Recife, Pernambuco, Brazil (8°03′35.4″ S, 34°59′41.6″ W). Access to the genetic heritage was registered in the Brazilian National System for the Management of Genetic Heritage and Associated Traditional Knowledge (SisGen) under registration number A08E18B. Plant material was collected by researchers from the Laboratory of Natural Products at the Federal University of Pernambuco (UFPE). Botanical identification was performed by Dr. Marccus Vinicius Alves, and a voucher specimen was deposited at the Professor Vasconcelos Sobrinho Herbarium (PEUFR), Federal University of Pernambuco, under accession number PEUFR35193.

### Extraction of *Piper aduncum* essential oil

Following collection, the leaves of *P. aduncum* were manually sorted to remove impurities and unwanted plant material. The selected leaves were subsequently washed, air-dried, and mechanically ground to facilitate essential oil extraction. The essential oil was obtained by hydrodistillation using a Clevenger-type steam distillation apparatus. Briefly, 2,058 g of ground leaves were mixed with distilled water at a 1:10 (w/v) ratio in a hydrodistillation flask and subjected to extraction for 5 h. After distillation, the essential oil was separated from the hydrolate and dried over anhydrous sodium sulfate (Na_2_SO_4_) to remove residual moisture. The resulting *Piper aduncum* essential oil (EO*Pa*) was stored in amber glass containers under appropriate conditions until use in the experimental assays.

### Phytochemical characterization of *Piper aduncum* essential oil

The chemical composition of EO*Pa* was characterized by gas chromatography coupled with mass spectrometry (GC–MS) using an Agilent Technologies 5975 C system (Palo Alto, CA, USA) equipped with a quadrupole mass detector and a non-polar DB-5 capillary column (Agilent J&W; 60 m × 0.25 mm i.d. × 0.25 μm film thickness).

Prior to analysis, the essential oil was diluted in hexane. Aliquots of 1 µL (2,000–3,000 ppm) were injected in split mode (split ratio 1:50). A homologous series of n-alkanes (C_9_–C_30_) was analyzed under identical chromatographic conditions for the determination of retention indices. Additionally, a mixture containing the essential oil and standard hydrocarbons was injected to facilitate constituent identification.

The oven temperature program was initiated at 60 °C and maintained for 3 min, followed by heating at 2.5 °C min^−1^ to 240 °C, where it was held for an additional 10 min. Helium was used as the carrier gas at a constant pressure of 100 kPa. The injector temperature was maintained at 250 °C, while the MS interface temperature was set at 200 °C. Mass spectra were acquired by electron ionization (EI) at 70 eV over an m/z range of 20–350.

Compound identification was achieved by comparing the obtained mass spectra with those available in the MassFinder 4, NIST08, and Wiley Registry™ (9th edition) databases using Agilent MSD Productivity ChemStation software. In addition, linear retention indices (LRIs) were calculated relative to the homologous series of n-alkanes and compared with published values reported by Adams (2007), thereby increasing the reliability of compound identification.

The relative quantification of the essential oil constituents was performed by gas chromatography with flame ionization detection (GC–FID) under the same chromatographic conditions employed for GC–MS analysis. All analyses were performed in triplicate, and the relative abundance of each constituent was calculated by peak-area normalization and expressed as the mean ± standard deviation.

### Experimental animals

Male Swiss albino mice (10–12 weeks old, 30–35 g) were obtained from the animal facility of the Keizo Asami Immunopathology Laboratory (LIKA), Federal University of Pernambuco (UFPE). Animals were housed in the Experimental Animal Facility of the Department of Biochemistry, UFPE, under controlled environmental conditions (22 ± 2 °C, 12 h light/12 h dark cycle), with free access to standard chow and water *ad libitum*. Following a one-week acclimatization period, the animals were weighed and randomly allocated to the experimental groups using a weight-balanced randomization procedure to ensure comparable body weights among groups. Treatment administration was not performed under blinded conditions, however, outcome assessment was conducted by an investigator blinded to the experimental group allocation.

The doses were selected based on preliminary experiments conducted in our laboratory, in which these doses provided a suitable range for representing low, intermediate, and high exposure levels. This dose range was therefore adopted to characterize the pharmacological response across increasing doses and to assess potential dose-dependent effects.

The pharmacological activities of EOPa were evaluated using complementary experimental models of pyrexia, inflammation, and nociception, based on previously established pharmacological approaches with minor modifications (Muhammad et al. 2012).

All experimental procedures were conducted in accordance with institutional guidelines for the care and use of laboratory animals and were approved by the Ethics Committee on Animal Use of the Federal University of Pernambuco (protocol no. 0121/2024).

### Brewer’s yeast-induced pyrexia

Pyrexia was induced according to the method described by Winter et al. (1962), with minor modifications. Initially, twenty-five mice were screened by measuring their basal rectal temperature. Subsequently, the animals received a subcutaneous injection of a 15% suspension of brewer’s yeast (*Saccharomyces cerevisiae*) prepared in physiological saline. Eighteen hours after yeast administration, only animals presenting a rectal temperature above 38.0 °C were included in the experiment.

The selected animals were randomly assigned to five groups (*n* = 5): negative control (0.9% saline, p.o.), positive control (dipyrone, 100 mg/kg, p.o.), and *Piper aduncum* essential oil (EO*Pa*; 50, 100, or 200 mg/kg, p.o.). For oral administration, EO*Pa* was dispersed in 0.9% saline solution and sonicated immediately before administration to promote homogeneous dispersion of the essential oil in the vehicle. The corresponding control groups received the same vehicle (0.9% saline solution). Rectal temperature was recorded before treatment and subsequently at 1-h intervals for 5 h following drug administration.

### Carrageenan-induced paw edema

Acute paw edema was induced by subplantar injection of 15 µL of 2% carrageenan solution into the right hind paw, following a modified version of the method described by Morris (2003). Animals were randomly divided into five groups (*n* = 5) and treated with negative control (0.9% saline), positive control (indomethacin, 20 mg/kg, i.p.), or EO*Pa* (50, 100, or 200 mg/kg, p.o.). One hour after treatment, all animals received carrageenan (20 mg/kg, intraplantar). For oral administration, EO*Pa* was dispersed where discredited previously.

Paw thickness was measured using a digital caliper at 1, 2, 3, 4, and 5 h after carrageenan injection. At the end of the experimental period, representative macroscopic photographs were obtained, and the animals were subsequently subjected to radiographic evaluation.

### Macroscopic and radiographic evaluation

At the end of the experimental protocol, animals were anesthetized with xylazine (2%, 1 mg/kg, i.p.) and ketamine (10%, 50 mg/kg, i.p.) and positioned in the prone position. Macroscopic photographs of the hind paws were acquired to document the extent of edema.

Radiographic images were subsequently obtained in the plantar projection using a Kodak X-ray system with a resolution of 2000 × 40 pixels, diaphragm aperture of 2.6 cm, and an exposure time of 60 s. Radiographs were analyzed for soft tissue swelling and changes in joint space architecture associated with the inflammatory process.

### Acetic acid-induced writhing test

Peripheral nociception was induced by intraperitoneal administration of 0.8% acetic acid at a volume of 0.1 mL/10 g body weight. Animals were randomly allocated into five groups (*n* = 5) and treated with negative control (0.9% saline, p.o.), positive control (indomethacin, 20 mg/kg, i.p.), or EO*Pa* (50, 100, or 200 mg/kg, p.o.). One hour after treatment, acetic acid was administered intraperitoneally. For oral administration, EO*Pa* was dispersed where discredited previously.

The number of abdominal writhes was recorded between 5 and 15 min after acetic acid injection according to Oliveira et al. (2018).

### Formalin test

The formalin-induced nociception test was performed according to Hunskaar and Hole (1987). Animals received an intraplantar injection of 20 µL of 2.5% formalin into the right hind paw. Mice were randomly divided into five groups (*n* = 5) and treated with negative control (0.9% saline, p.o.), positive controls (indomethacin, 20 mg/kg, i.p., and morphine, 10 mg/kg, i.p.), or EO*Pa* (50, 100, or 200 mg/kg, p.o.). For oral administration, EO*Pa* was dispersed where discredited previously.

Nociceptive behavior was quantified by measuring the total time spent licking or biting the injected paw. Two distinct phases of nociceptive behavior were evaluated: the neurogenic phase (0–5 min after formalin injection) and the inflammatory phase (15–30 min after formalin administration).

### Investigation of the antinociceptive mechanism of action

To investigate the mechanisms underlying the antinociceptive activity of EO*Pa*, separate groups of mice were pretreated with the following pharmacological antagonists: naloxone (2 mg/kg, i.p.), atropine (1 mg/kg, i.p.), glibenclamide (5 mg/kg, i.p.), or N-ω-nitro-L-arginine methyl ester (L-NAME, 5 mg/kg, i.p.). Fifteen minutes after antagonist administration, animals received EO*Pa* (200 mg/kg, p.o.) or 0.9% saline (p.o.). For oral administration, EO*Pa* was dispersed where discredited previously.

The formalin test was subsequently performed as described above, and nociceptive responses were evaluated during both the neurogenic and inflammatory phases to determine the involvement of opioid receptors, muscarinic receptors, ATP-sensitive potassium (K^+^ _ATP_) channels, and nitric oxide synthesis in the antinociceptive effects of EO*Pa*.

### Statistical analysis

All in vivo experimental data are presented as the mean ± standard deviation (SD). Statistical analyses were performed using GraphPad Prism version 8.0 (GraphPad Software, San Diego, CA, USA). Comparisons between groups were performed using one-way or two-way analysis of variance (ANOVA), depending on the test, followed by Tukey’s post hoc test for multiple comparisons. Statistical significance was set at *p* < 0.05.

## Results and discusion

### Essential oil yield and chemical composition

**Fig. 1:**
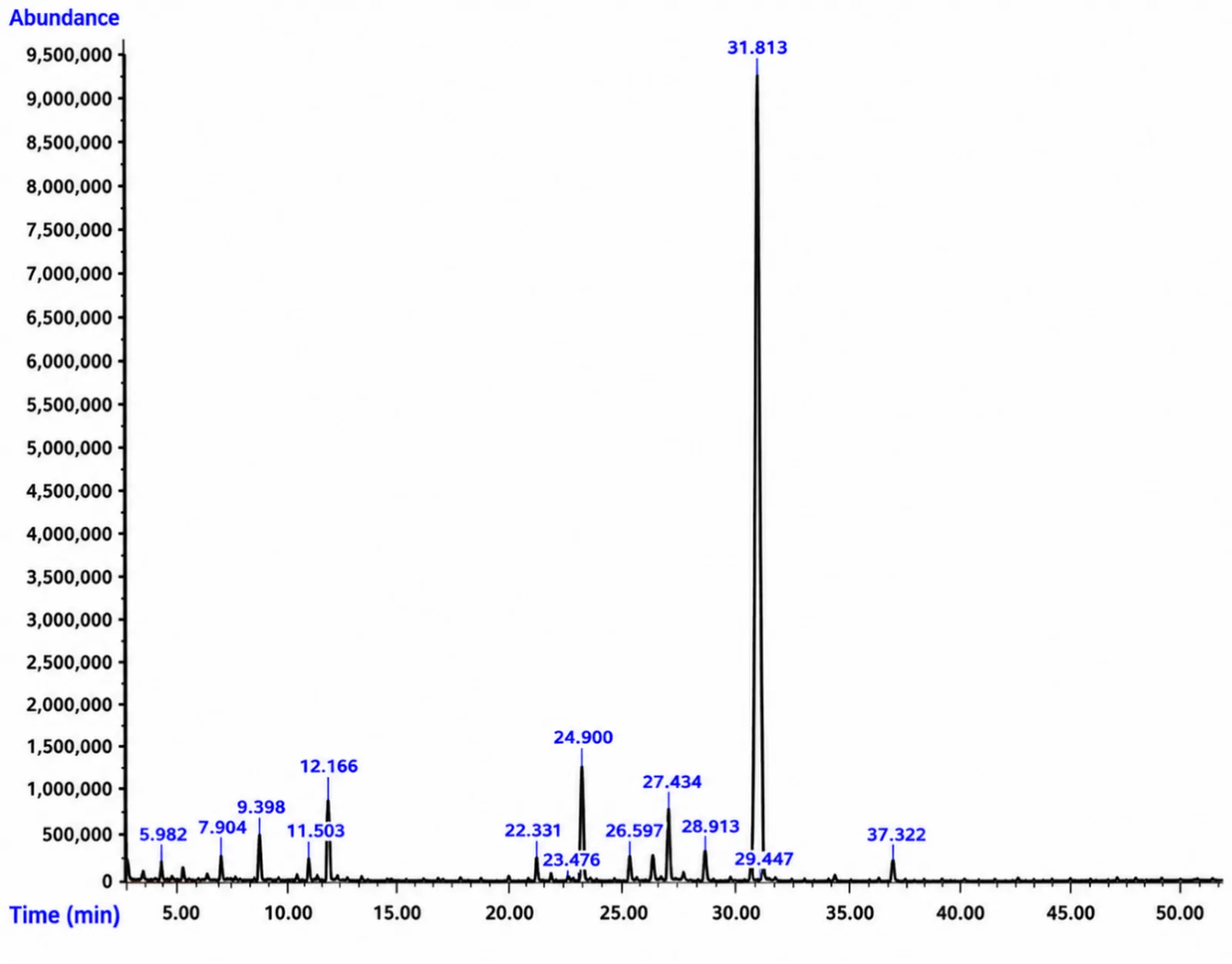
– GC–MS/GC-FID chromatogram of *Piper aduncum* essential oil (EO*Pa*). Each peak represents the retention time associated with a specific compound

Hydrodistillation of *Piper aduncum* leaves yielded of 0.83% (w/w) the essential oil, which was subsequently characterized by gas chromatography–mass spectrometry (GC–MS) and gas chromatography with flame ionization detection (GC–FID). The chromatographic profile is presented in Fig. 1, and the identified constituents are summarized in the corresponding histogram. Dilapiol was identified as the predominant constituent, accounting for 69.65 ± 2.12% of the total essential oil composition.

The chemical profile of EO*Pa* is characterized by the predominance of dilapiol, a phenylpropanoid biosynthesized through the shikimate pathway. Following the formation of the aromatic amino acid’s phenylalanine and tyrosine, a series of enzymatic reactions involving reduction, oxidation, and cyclization gives rise to different phenylpropanoids. This process is initiated by the enzyme phenylalanine ammonia-lyase (PAL), which catalyzes the first step of the phenylpropanoid pathway through cinnamic acid formation (Martin et al. 2023). PAL expression is markedly upregulated under environmental stress conditions, particularly excessive ultraviolet radiation, leading to enhanced phenylpropanoid biosynthesis and consequently increased accumulation of dilapiol in *P. aduncum* (Wang et al. 2020).

Previous studies have demonstrated that solar radiation is one of the major environmental factors regulating dilapiol biosynthesis in *P. aduncum* (Assunção et al. 2023; Pacheco et al. 2016). This phenylpropanoid is believed to contribute to plant adaptation by participating in physiological processes associated with water balance and protection against abiotic stress. Owing to its high abundance, dilapiol has attracted considerable attention because of its diverse pharmacological properties, particularly its anti-inflammatory activity (Parise-Filho et al., 2011), in addition to promising effects against diabetic nephropathy (Shafi, Khurana, & Gupta, 2025).

### Brewer’s yeast-induced pyrexia

**Fig. 2:**
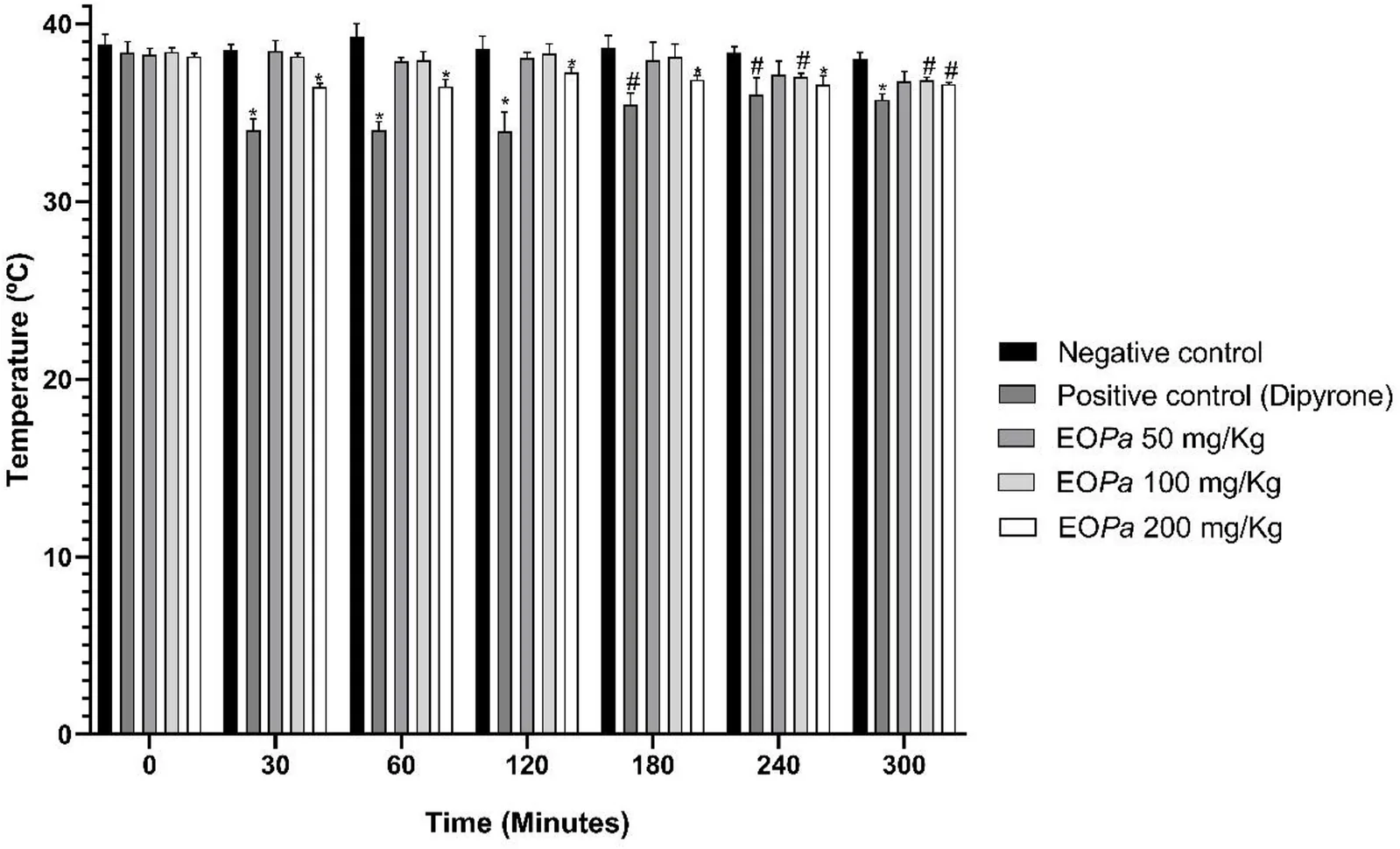
Antipyretic effect of OE*Pa* evaluated by rectal temperature measurements. Data are presented as means ± standard deviation (SD) (*n* = 5), analyzed by two-way ANOVA followed by Tukey’s multiple comparisons test. (*) *p* < 0.001 and (#) *p* < 0.01 versus the negative control

The antipyretic activity of EO*Pa* is presented in Fig. 2, demonstrating its ability to reduce fever induced by *Saccharomyces cerevisiae*. Among the evaluated doses, EO*Pa* at 200 mg/kg produced the most pronounced effect, significantly reducing body temperature within the first 30 min after treatment.

At this dose, inhibition of fever reached 46.0%, 53.0%, 54.1%, 55.2%, 76.1%, and 62.3% at 30, 60, 120, 180, 240, and 300 min, respectively. EO*Pa* administered at 100 mg/kg exhibited significant antipyretic activity only after 240 min, reducing body temperature by 57.8%. In contrast, the 50 mg/kg dose did not produce a statistically significant antipyretic effect throughout the observation period.

The positive control, dipyrone (100 mg/kg), produced a rapid and complete antipyretic response, abolishing fever within the first 30 min after treatment and maintaining normothermia for approximately 120 min. Thereafter, body temperature gradually increased, indicating recurrence of the febrile response.

Administration of brewer’s yeast (*Saccharomyces cerevisiae*) triggered a systemic inflammatory response characterized by increased body temperature as part of the host defense against the inflammatory stimulus (Kulkarni et al., 2025). This response is associated with elevated production of pyrogenic mediators, including interleukin-6 (IL-6), interleukin-1β (IL-1β), tumor necrosis factor-α (TNF-α), prostaglandin E_2_ (PGE_2_), cyclic adenosine monophosphate (cAMP), and cyclooxygenase-2 (COX-2) (Li et al., 2025). These mediators ultimately increase the hypothalamic thermoregulatory set point, resulting in fever development (Blomqvist and Engblom 2018).

Dipyrone exerts its antipyretic effect primarily through inhibition of prostaglandin synthesis by suppressing cyclooxygenase activity, thereby reducing hypothalamic PGE₂ production. Consequently, endogenous pyrogenic cytokines, including IL-1β, TNF-α, and IL-6, are no longer able to sustain the elevated thermoregulatory set point (Kefa et al., 2025).

Similarly, EO*Pa* exhibited significant antipyretic activity, particularly at 200 mg/kg. Nevertheless, both EO*Pa* and dipyrone showed progressive loss of efficacy over time, most likely because of drug metabolism and elimination while the inflammatory stimulus remained active. As the infectious stimulus persisted, continuous production of pyrogenic mediators, especially PGE₂, maintained hypothalamic thermoregulatory dysregulation, explaining the recurrence of hyperthermia observed at later time points (Haidar and Singh 2022; Roth; De Souza, 2001).

### Carrageenan-induced paw edema

**Fig. 3:**
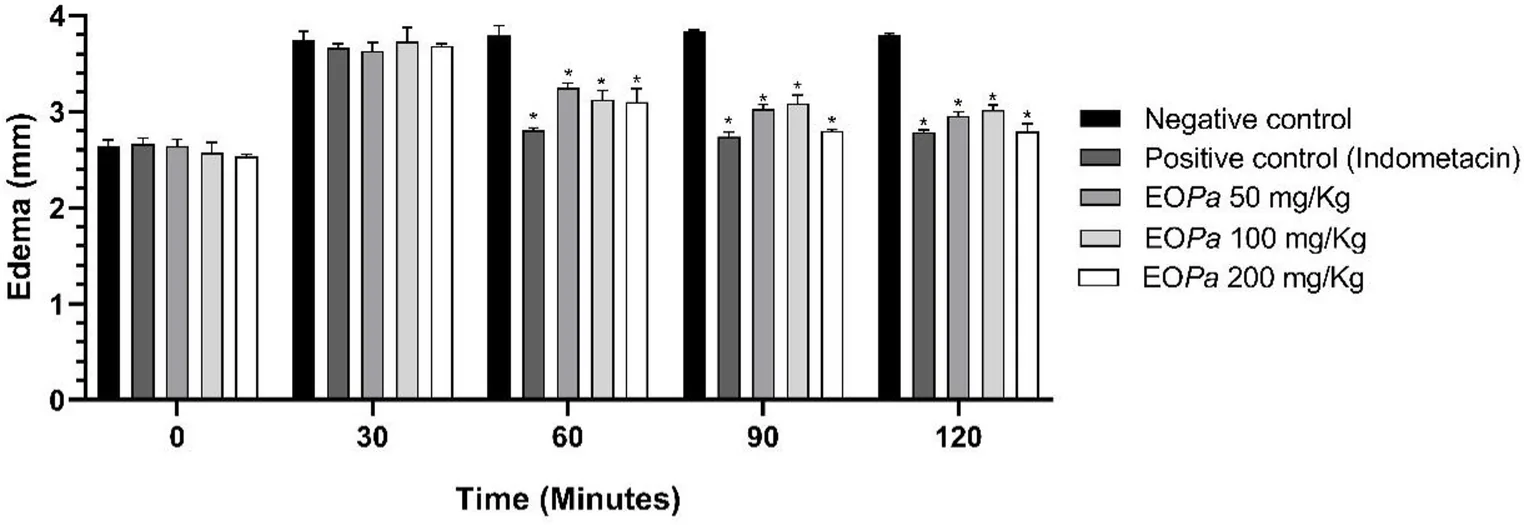
– Edema-inhibitory effect of OE*Pa* based on paw volume measurements. Data are presented as means ± standard deviation (SD) (*n* = 5), analyzed by ANOVA followed by Tukey’s multiple comparisons test. (*) Indicates significant differences (*p* < 0.0001) compared with the negative control

The anti-inflammatory effect of EO*Pa* in the carrageenan-induced paw edema model is shown in Fig. 3. EO*Pa* significantly reduced paw swelling at all tested doses throughout the experimental period, demonstrating marked anti-edematogenic activity.

During the first hour after carrageenan administration, edema inhibition reached 25.92% in the indomethacin-treated group, whereas EO*Pa* reduced paw swelling by 14.24%, 17.54%, and 18.20% at doses of 50, 100, and 200 mg/kg, respectively. At 90 min, edema inhibition increased to 28.75% for indomethacin and 21.01%, 19.31%, and 26.89% for EO*Pa* at the respective doses. At 120 min, inhibition values were 26.61% for indomethacin and 22.13%, 21.48%, and 26.48% for EO*Pa*, indicating that the highest EO*Pa* dose produced an anti-inflammatory effect comparable to that of the reference drug.

Edema formation is one of the hallmark features of acute inflammation and results primarily from increased vascular permeability, allowing protein-rich exudate to migrate from the intravascular compartment into the surrounding interstitial tissue (Soliman and Barreda 2022). The present findings demonstrate that EO*Pa* effectively attenuated edema formation at all evaluated doses, with the 200 mg/kg treatment exhibiting efficacy statistically comparable to indomethacin.

Carrageenan-induced inflammation is characterized by a biphasic response. The early phase involves the release of histamine, serotonin, and bradykinin, which promote vasodilation, increased vascular permeability, edema formation, and leukocyte recruitment. The late phase is predominantly mediated by prostaglandins, particularly PGE₂, together with pro-inflammatory cytokines such as TNF-α, IL-1β, and IL-6, which sustain the inflammatory response (Sofidiya et al., 2025; Souza; Casais-e-Silva; Aguiar, 2020).

Indomethacin, a non-steroidal anti-inflammatory drug (NSAID), exerts its pharmacological activity mainly by inhibiting cyclooxygenase enzymes, particularly COX-1, thereby preventing the conversion of arachidonic acid into prostaglandins (Saleem; Mohammed; Al-Taee, 2025). Consequently, prostaglandin-dependent inflammatory signaling is suppressed, reducing leukocyte recruitment, vascular permeability, and tissue edema (Silva et al., 2026).

Although indomethacin is highly effective in controlling inflammation, its long-term clinical use is limited by significant adverse effects, including renal dysfunction, gastrointestinal bleeding and ulceration, intestinal perforation, and impaired platelet function (Sutton et al., 2024). These limitations reinforce the importance of identifying novel anti-inflammatory agents from natural products that exhibit comparable efficacy while presenting a more favorable safety profile.

### Radiographic evaluation

**Fig. 4:**
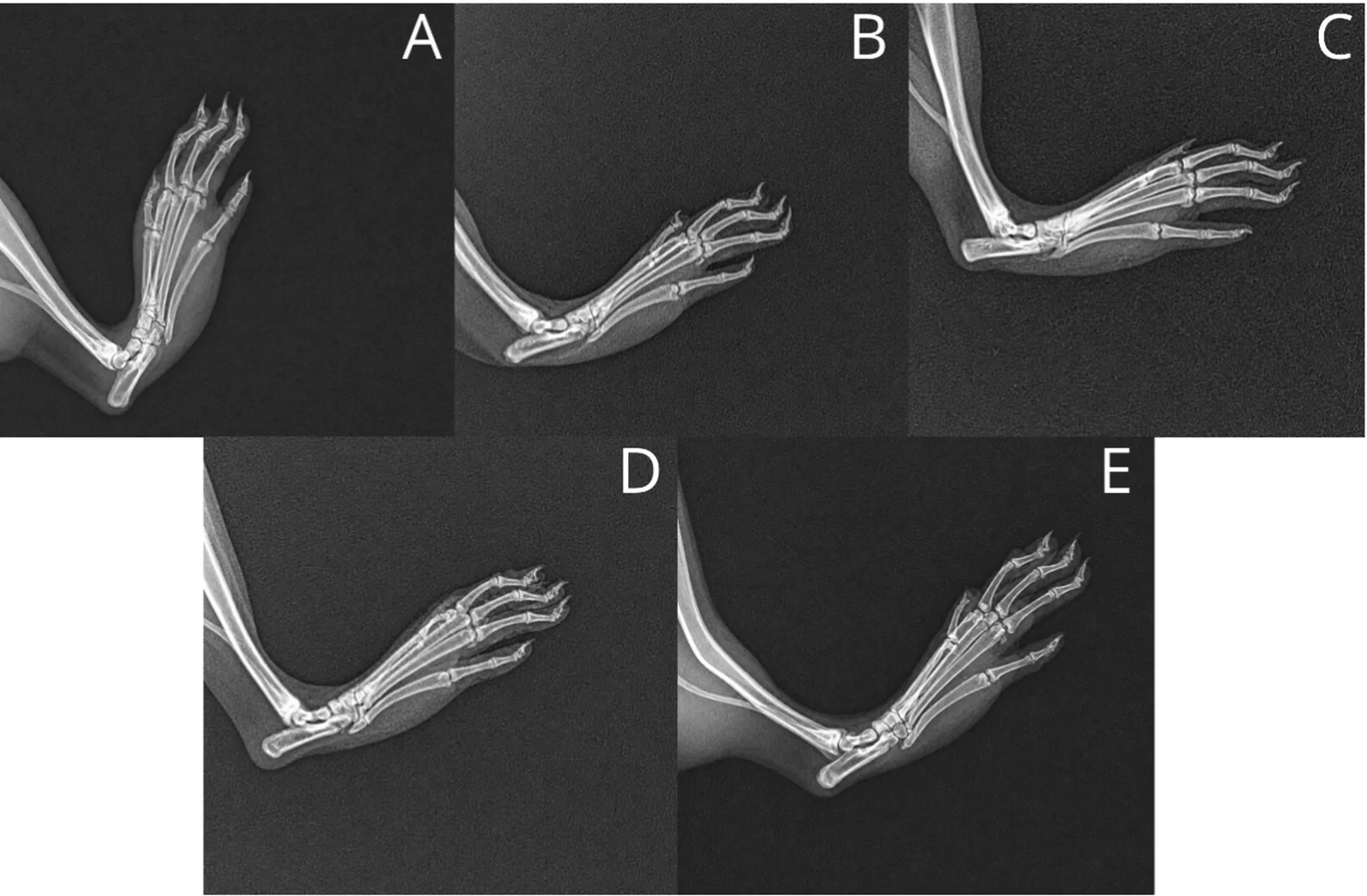
Radiographic image obtained following the carrageenan-induced edema test. **A** Negative control (0.9% NaCl); **B** Positive control (Indomethacin 20 mg/kg); **C** EO***Pa*** (50 mg/kg); **D** EO*Pa* (100 mg/kg); **E** EO*Pa* (200 mg/kg)

The radiographic findings provided complementary evidence of the acute inflammatory response observed in the paw edema test. Radiographs (Fig. 4) obtained from the negative control group showed increased soft-tissue thickness, evidenced by a greater distance between the dermal tissue and the underlying bone structures, consistent with edema formation and accumulation of inflammatory exudate. A reduction in the apparent joint space was also observed, which may be associated with compression of adjacent structures resulting from tissue swelling. Conversely, animals treated with either indomethacin (Fig. 4 (B) or EO*Pa* (Fig. 4 (C-E) showed attenuation of these radiographic alterations, characterized primarily by reduced soft-tissue thickening.

The effect was more evident at the highest EO*Pa* dose (200 mg/kg), whose radiographic appearance was comparable to that observed in the indomethacin-treated group. These findings provide complementary imaging evidence of the anti-inflammatory effect of EO*Pa* in the carrageenan-induced acute paw edema model. These results reinforce the anti-inflammatory activity of EO*Pa* and are consistent with previous studies demonstrating that natural products with anti-inflammatory properties effectively reduce soft tissue edema and mitigate morphological alterations detected by macroscopic and radiographic analyses (Katare et al. 2025; Semis et al. 2021).

### Acetic acid-induced writhing test

**Fig. 5:**
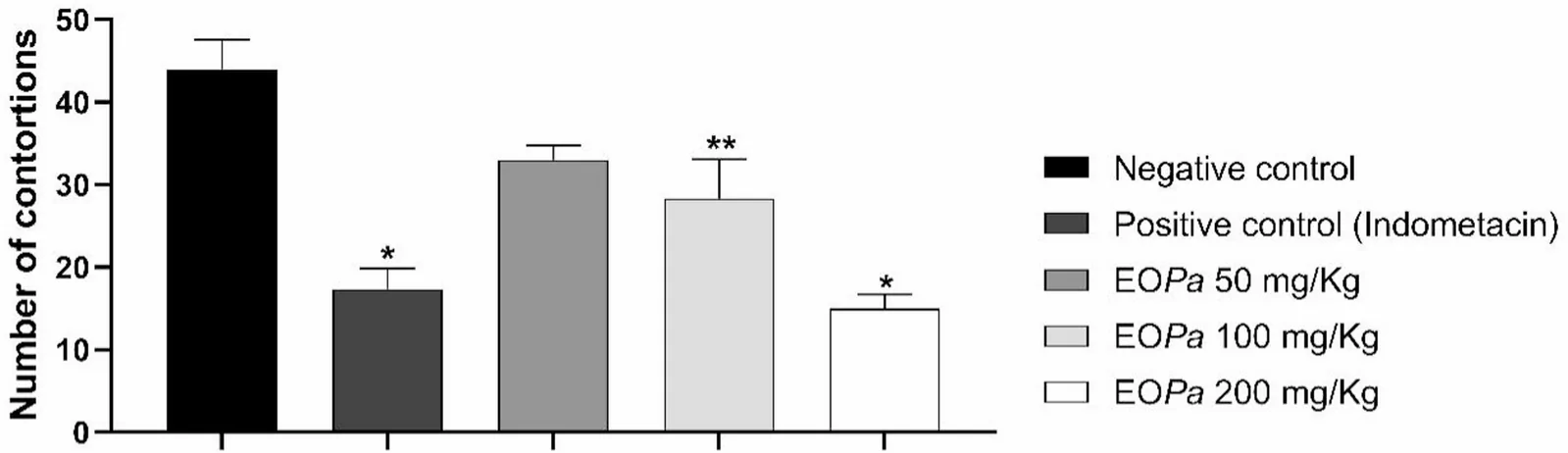
– Antinociceptive effect of OE*Pa* based on the number of abdominal writhes. Data are presented as means ± standard deviation (SD) (*n* = 5), analyzed by on-way ANOVA followed by Tukey’s multiple comparisons test. (*) Indicates significant differences (*p* < 0.001) and (**) Indicates significant differences (*p* < 0.01) compared with the negative control

The results of the acetic acid-induced writhing test are presented in Fig. 5, demonstrating the antinociceptive activity of EO*Pa*. The highest dose evaluated (200 mg/kg) produced the greatest analgesic effect, reducing the number of abdominal writhes by 65.90% compared with the negative control group. This reduction was statistically superior to those observed at the lower doses.

The positive control (indomethacin, 20 mg/kg) inhibited abdominal writhing by 60.61%, whereas EO*Pa* at 50 and 100 mg/kg reduced nociceptive behavior by 25.00% and 35.61%, respectively.

The acetic acid-induced writhing assay is a well-established model of inflammatory pain widely employed to screen analgesic and anti-inflammatory agents, particularly compounds derived from natural products (Dadan Junior et al., 2023). The present findings demonstrate that EO*Pa* exerts a dose-dependent antinociceptive effect, with increasing efficacy as the administered dose increased. Notably, the 200 mg/kg dose produced analgesic activity statistically comparable to that of indomethacin, indicating potent peripheral antinociceptive activity.

Intraperitoneal administration of acetic acid induces nociception by stimulating the production of cyclooxygenase (COX) and lipoxygenase (LOX)-derived metabolites, resulting in the release of endogenous inflammatory mediators such as prostaglandins, bradykinin, serotonin, histamine, and pro-inflammatory cytokines (Machado et al., 2025). Moreover, tissue acidosis produced by acetic acid activates acid-sensing ion channels (ASICs) and transient receptor potential vanilloid 1 (TRPV1) receptors, promoting activation of peripheral nociceptors and generating the characteristic pain response (Hung et al. 2023). Therefore, the significant inhibition of abdominal writhing observed following EO*Pa* treatment suggests that the essential oil interferes with inflammatory mediator production and/or nociceptor activation.

### Formalin Test

**Fig. 6:**
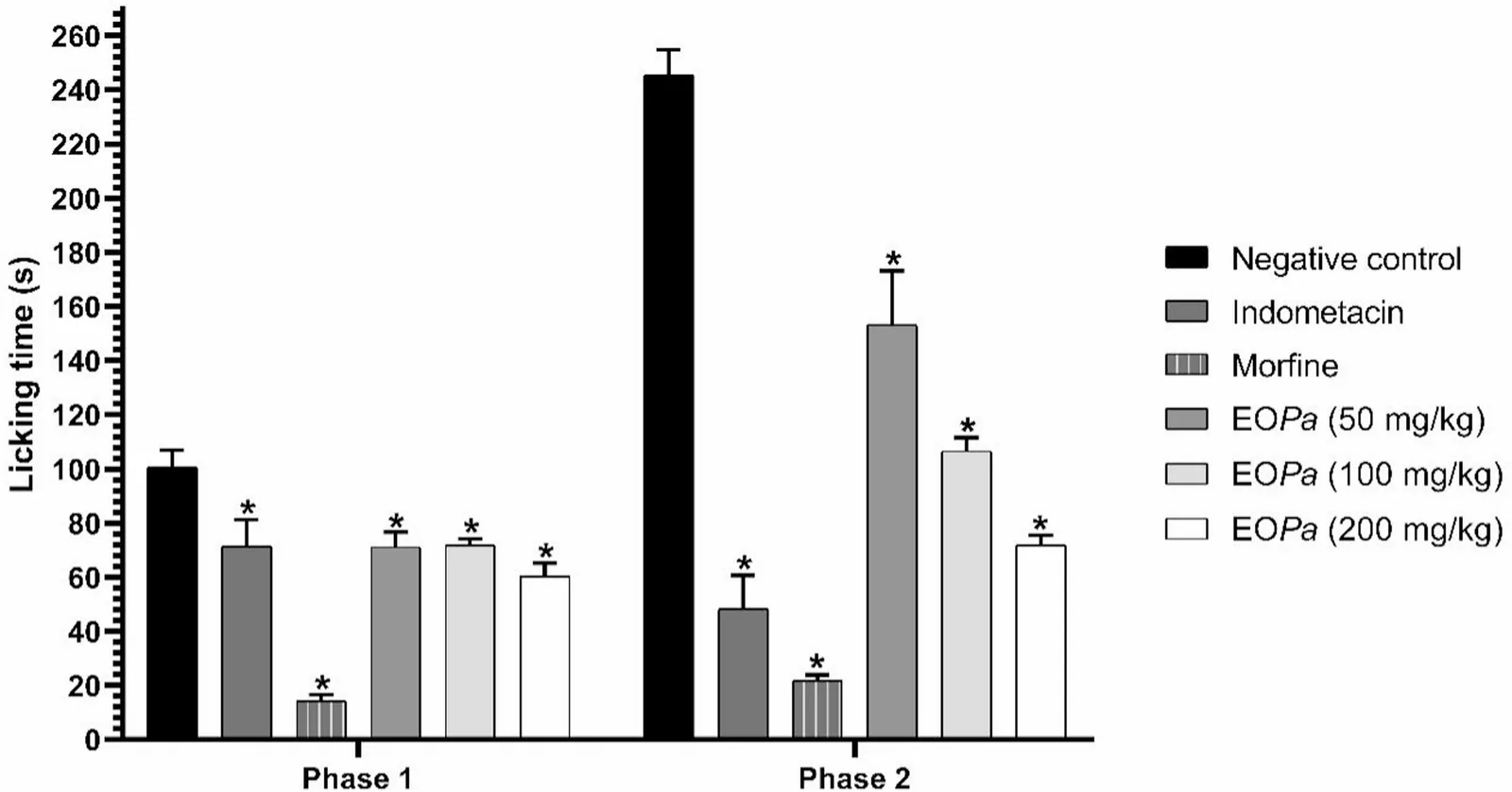
– Antinociceptive effect and mechanisms of action of the essential oil from *Piper aduncum* by induction with formalin (i.pl.), in the neurogenic phase (phase 1) and inflammatory phase (phase 2). Data are presented as means ± standard deviation (SD) (*n* = 5), analyzed by on-way ANOVA followed by Tukey’s multiple comparisons test. (*) *p* < 0.0001 versus control

Figure 6 illustrates the antinociceptive activity of EO*Pa* during both phases of the formalin test. During the first (neurogenic) phase, EO*Pa* reduced nociceptive behavior by 29.17%, 28.42%, and 39.89% at doses of 50, 100, and 200 mg/kg, respectively. Under the same conditions, indomethacin produced 29.02% inhibition, whereas morphine inhibited nociceptive behavior by 85.68%.

Although EO*Pa* significantly reduced neurogenic pain compared with the negative control group, its effect was considerably lower than that of morphine, suggesting only modest central antinociceptive activity. The first phase of the formalin test reflects direct activation of peripheral nociceptors through chemical stimulation of myelinated Aδ fibers and unmyelinated C fibers (Fernandes et al., 2025). Consequently, the moderate effect observed during this phase suggests that opioid-mediated mechanisms are unlikely to represent the primary pathway responsible for the antinociceptive activity of EO*Pa*.

During the second (inflammatory) phase, EO*Pa* inhibited nociceptive behavior by 37.57%, 56.55%, and 70.67% at doses of 50, 100, and 200 mg/kg, respectively. Indomethacin and morphine produced inhibition rates of 80.28% and 70.67%, respectively.

The inflammatory phase develops after the neurogenic phase and depends on the local release of inflammatory mediators, particularly prostaglandins and leukotrienes, which sensitize peripheral nociceptors and sustain pain transmission (Fernandes et al., 2025). The marked efficacy of EO*Pa* during this phase, together with its pharmacological profile resembling that of indomethacin, strongly suggests that its antinociceptive activity is predominantly associated with inhibition of inflammatory mediator production rather than direct modulation of central nociceptive pathways.

### Investigation of the antinociceptive mechanism

Table 1, regarding the mechanistic studies demonstrated that pretreatment with atropine or glibenclamide almost completely abolished the antinociceptive activity of EO*Pa* during both the neurogenic and inflammatory phases of the formalin test. Animals pretreated with atropine exhibited no inhibition of nociceptive behavior (0%), whereas glibenclamide reduced EO*Pa* activity to only 2.08% inhibition, values comparable to those observed in the saline-treated group. In contrast, pretreatment with naloxone or L-NAME did not significantly modify the antinociceptive effect of EO*Pa*, indicating that opioid receptors and nitric oxide synthesis are unlikely to play major roles in its mechanism of action.

The blockade produced by atropine indicates that muscarinic cholinergic receptors are critically involved in the analgesic activity of EO*Pa*. Muscarinic acetylcholine receptors (mAChRs) are widely distributed throughout both the peripheral and central nervous systems and play a fundamental role in pain modulation (Grauer et al., 2020). Although atropine does not distinguish among receptor subtypes, its ability to abolish EO*Pa*-induced antinociception strongly.

**Table 2:** Activity of antinociceptive pathways of *Piper aduncum* essential oil by induction with formalin. Data are presented as means ± S.D. (*n* = 5). (*) Indicates a statistically significant difference compared to the negative control group, difference (*p* < 0.001), by on-way ANOVA followed by Tukey’s multiple comparisons test

Mechanism	Pretreatment	Dose (mg/kg; i.*p*.)	Treatment	Dose (mg/kg; *p*.o.)	Licking time - Phase 1 (seg)	Licking inhibition - Phase 1 (%)	Licking time - Phase 2 (seg)	Licking inhibition - Phase 2 (%)
Control	Saline	–	Saline	–	100.6	–	245.5	–
	Saline	–	EO*Pa*	200	60.5	39.86*	164.67	32.92*
Receptores Muscarínicos Antagonist Blocker	Atropine	1	Saline	–	96.2	–	250.5	–
	Atropine	1	EO*Pa*	200	101.33	0.0	265.0	0.0
ATP-dependent potassium channels (K^+^ _ATP_) blocker	Glibenclamide	5	Saline	–	102.4	–	216.67	–
	Glibenclamide	5	EO*Pa*	200	98.5	2.08	253.67	0.0
Opioid receptors antagonist	Naloxone	2	Saline	–	102.6	–	250.33	–
	Naloxone	2	EO*Pa*	200	68.6	31.80*	124.0	49.49*
NO synthase inhibitor	L-NAME	5	Saline	–	101.0	–	279.0	–
	L-NAME	5	EO*Pa*	200	65.0	35.38*	151.33	38.35*

suggests activation of muscarinic signaling pathways. Previous studies have demonstrated that direct activation of mAChRs produces robust antinociceptive effects, highlighting the importance of cholinergic neurotransmission in pain regulation (Montigné & Balayssac, 2023).

The involvement of muscarinic receptors was also evident during the inflammatory phase. Activation of these receptors has been associated with increased intracellular Ca^2+^ concentrations and enhanced gene expression in T and B lymphocytes, indicating that cholinergic signaling also participates in the regulation of immune cell function (Calzetta et al., 2022). Among the muscarinic receptor subtypes, M3 receptors are of particular interest because acetylcholine-mediated activation of endothelial M3 receptors stimulates nitric oxide production, promoting vascular smooth muscle relaxation and vasodilation. This response facilitates tissue perfusion and immune cell trafficking to sites of inflammation, thereby contributing to the modulation of inflammatory processes (Mashimo et al., 2021).

Pretreatment with glibenclamide likewise abolished the antinociceptive effect of EO*Pa* during both phases of the formalin test, indicating the involvement of ATP-sensitive potassium (K^+^ _ATP_) channels. Activation of these channels promotes potassium efflux, resulting in membrane hyperpolarization and reduced neuronal excitability. Consequently, the generation of action potentials and transmission of nociceptive signals are suppressed, leading to antinociception. The ability of glibenclamide to reverse the analgesic effect of EO*Pa* therefore suggests that activation of K^+^ _ATP_ channels constitutes an important component of its mechanism of action (Jabbari et al. 2025; Izquierdo et al. 2013).

## Conclusion

The present findings demonstrate that the essential oil of *Piper aduncum* exhibits significant pharmacological activity against the principal manifestations of acute inflammation, producing marked reductions in pain, edema, and fever across the experimental models employed. The most pronounced effects were observed at the highest tested dose (200 mg/kg), indicating a dose-dependent response and suggesting greater therapeutic efficacy at this concentration. Mechanistic investigations further suggest that the anti-inflammatory and antinociceptive effects of the essential oil are mediated, at least in part, through modulation of inflammatory mediators and activation of cholinergic muscarinic receptors and ATP-sensitive potassium (K^+^ _ATP_) channels, both of which play key roles in nociceptive signaling. These findings provide new insights into the pharmacological mechanisms underlying the biological activity of *P. aduncum* essential oil. Overall, the results highlight the therapeutic potential of *P. aduncum* as a promising natural source of bioactive compounds for the development of novel anti-inflammatory and analgesic agents. Nevertheless, further studies are required to fully elucidate its molecular mechanisms of action, identify the constituents responsible for its pharmacological effects, and establish its efficacy and safety through additional preclinical and clinical investigations.

## Data Availability

No datasets were generated or analysed during the current study.
